# Nanoparticle Size Threshold for Magnetic Agglomeration and Associated Hyperthermia Performance

**DOI:** 10.3390/nano11112786

**Published:** 2021-10-21

**Authors:** David Serantes, Daniel Baldomir

**Affiliations:** Instituto de Investigacións Tecnolóxicas and Applied Physics Department, Universidade de Santiago de Compostela, 15782 Santiago de Compostela, Spain; daniel.baldomir@usc.es

**Keywords:** magnetic nanoparticles, magnetic agglomeration, magnetic hyperthermia

## Abstract

The likelihood of magnetic nanoparticles to agglomerate is usually estimated through the ratio between magnetic dipole-dipole and thermal energies, thus neglecting the fact that, depending on the magnitude of the magnetic anisotropy constant (*K*), the particle moment may fluctuate internally and thus undermine the agglomeration process. Based on the comparison between the involved timescales, we study in this work how the threshold size for magnetic agglomeration ($d_{aggl}$) varies depending on the *K* value. Our results suggest that small variations in *K*-due to, e.g., shape contribution, might shift $d_{aggl}$ by a few nm. A comparison with the usual *superparamagnetism* estimation is provided, as well as with the energy competition approach. In addition, based on the key role of the anisotropy in the hyperthermia performance, we also analyse the associated heating capability, as non-agglomerated particles would be of high interest for the application.

## 1. Introduction

Based on the possibility to achieve local actuation by a harmless remote magnetic field, magnetic nanoparticles (MNPs) are very attractive candidates for novel medical applications [1,2]. Particularly iron oxides, based on their good biocompatibility [3], have been the subject of intense research in recent years, for example for magnetic hyperthermia cancer therapy [4,5] or drug release [6,7]. A key aspect defining the performance of the MNPs under external magnetic fields is their magnetic anisotropy, as it allows them to transform the absorbed electromagnetic energy into the required physical stimuli to promote specific cell behaviours, acting, in practice, as medical nanorobots [8].

Another key aspect to consider when dealing with magnetic nanoparticles for biomedical applications is the agglomeration likelihood, as it could affect not only the metabolising process but also the magnetic properties by changing the interparticle interactions [9]. Considering for example magnetic hyperthermia, it is known that the particles tend to agglomerate when internalized by the cells and such may lead to a decrease of the heating performance [10]. However, the opposite behaviour has also been reported, with an increase of the heat release if the particles form chains [11]. The problem is that while accounting for the effect of interparticle dipolar interactions is of primary importance for a successful application [12], the usual estimate of agglomeration likelihood, i.e., the ratio between the dipolar energy of parallel-aligned moments and thermal energy [13,14],

(1) $$\Gamma =\frac{\mu_{0}{\left(M_{S}V\right)}^{2}}{2\pi {l_{cc}}^{3}k_{B}T},$$

in the limit case of touching particles (i.e., $l_{cc}=d$), does not consider the magnetic anisotropy despite its key role in governing the magnetisation behaviour. In Equation (1), $\mu_{0}=1.256\times 10^{-6}$ Tm/A is the permeability of free space, $M_{S}$ the saturation magnetisation, and $l_{cc}$ the center to center interparticle distance. In this work we suggest an approach to consider the magnetic anisotropy into the agglomeration likelihood based on the comparison of characteristic relaxation times.

The complex role of the interparticle interactions often prompts researchers to the use of *superparamagnetic* (SPM) particles, with the idea that the rapid internal fluctuation of the particles’ magnetic moments shall prevent their agglomeration. Thus, at the first approximation one could be tempted to consider that agglomeration will not occur for particles with blocking temperature ($T_{B}$) below the desired working temperature, since for $T>T_{B}$ the particles are in the SPM state (i.e., they behave as giant paramagnetic-like *supermoments*). However, it must be kept in mind that behaving SPM-like is not an absolute term, but it is defined by the experimental timescale. Thus, regarding agglomeration, a particle could be referred to as SPM if its Néel relaxation time, $\tau_{N}$, is smaller than the characteristic timescales that allow agglomeration, i.e., diffusion ($\tau_{diff}$) and rotation ($\tau_{B}$) [15]. For the simplest case of uniaxial anisotropy, $\tau_{N}$ can be estimated as [16]

(2) $$\tau_{N}=\frac{\sqrt{\pi }}{2}\tau_{0}\frac{e^{\frac{KV}{k_{B}T}}}{{\left(\frac{KV}{k_{B}T}\right)}^{\frac{1}{2}}},$$

where the prefactor $\tau_{0}$ usually ranges between $10^{-9}$ and $10^{-12}$ s, *K* is the uniaxial anisotropy constant, *V* the particle volume, and $k_{B}$ the Boltzmann constant. The diffusion time can be expressed as

(3) $$\tau_{diff}=\frac{{\langle x\rangle }^{2}6\pi \eta R_{hyd}}{k_{B}T},$$

where ${\langle x\rangle }^{2}$ is the mean square displacement for a translating Brownian particle [17], η the viscosity of the embedding media, and $R_{hyd}$ is the hydrodynamic radius, defined by the particle size plus a nonmagnetic coating of thickness $t_{nm}$. For simplicity we consider spherical particles of diameter *d*. The rotation time $\tau_{B}$ (also referred as Debye [18] or Brownian time [19]) is expressed as

(4) $$\tau_{B}=\frac{3\eta V_{hyd}}{k_{B}T},$$

where $V_{hyd}$ is the hydrodynamic volume. Extensive details about the different relaxation mechanisms can be found in Coffey et al. [20].

The objective of this work is to estimate the size threshold for magnetic agglomeration, $d_{aggl}$ (i.e., size for which $\tau_{N}>\tau_{diff},\tau_{B}$, so that agglomeration is likely) in terms of *K*. Focusing on magnetite-like parameters based on its primary importance for bioapplications, we will consider different *effectiveK* values, which can be ascribed to dominance of shape anisotropy over the magnetocrystalline one [21,22]. Comparison will be made with the usual estimate of agglomeration likelihood, Equation (1). Then, the hyperthermia properties for the obtained $d_{aggl}$ will be studied. It must be recalled here the double role of *K* in the heating performance, as it determines both the maximum achievable heating [23,24] and the effectiveness in terms of field amplitude [25]; for completeness, this double role of *K* will also be briefly summarized. Please note that we are using “agglomeration” referring to a *reversible* process, distinct from the *irreversible* “aggregation” [26].

## 2. Results and Discussion

### 2.1. Size Threshold for Magnetic Agglomeration, $d_{Aggl}$

To estimate $d_{aggl}$ we followed the same approach as we did in Ref. [15]: to compare the characteristic Néel, diffusion, and rotation times, to obtain $d_{aggl}$ as the size for which $\tau_{N}>\tau_{diff},\tau_{B}$. In Equations (3) and (4) we have at first set $t_{nm}=0$, and used η=0.00235 kg/m·s, as in Ref. [19], which is comparable to that of HeLa cells for nm-scale dimensions [27]. We considered three cases for Equation (2): K=8,11, and 15 kJ/m${}^{3}$, i.e., values of the order found in the literature for magnetite particles [15,28,29]. The diffusion distance in Equation (3) is set as the interparticle distance at which the magnetostatic energy dominates over the thermal one, i.e., Γ>1 [15], so that:

(5) $$\langle x\rangle ={\left(\frac{\mu_{0}{\left(M_{S}V\right)}^{2}}{2\pi k_{B}T}\right)}^{\frac{1}{3}}.$$

**Figure 1:**
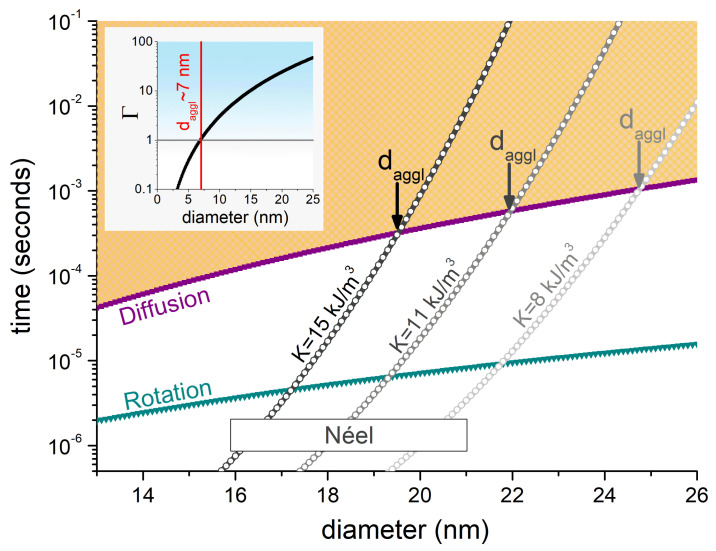
Diffusion ($\tau_{diff}$; purple line), rotation ($\tau_{B}$; green line), and Néel relaxation time ($\tau_{N}$; grey lines), as a function of the particle diameter. The distinct $\tau_{N}$ curves correspond to the different *K* values indicated. The dashed light-orange area indicates the range where agglomeration can be expected. The inset shows the size dependence of the Γ, which predicts agglomeration for sizes d>7 nm.

Note that while we have chosen Γ=1 to have a well defined criterion, agglomeration usually requires higher Γ values [30]. That is to say, we are searching for the lower $d_{aggl}$ boundary. With the same spirit, in Equation (1) we used $M_{S}=4.8\times 10^{5}$ A/m, i.e., the upper value for magnetite so that the interaction is, most likely, overestimated. The relaxation times as a function of the particle size are shown in Figure 1.

In Figure 1 it is clearly observed how increasing *K* leads to more stable moments, thus favouring agglomeration at smaller sizes (from $d_{aggl}∼25$ nm for K=8 kJ/m${}^{3}$, to $d_{aggl}∼20$ nm for K=15 kJ/m${}^{3}$). The inset shows the size dependence of Γ, which i) does not distinguish among particle characteristics (in terms of *K*, as previously mentioned), and; ii) predicts the dominance of the dipolar energy for much smaller particle sizes, with $d_{aggl}∼7$ nm. It is worth noting that the threshold value obtained for the K=11 kJ/m${}^{3}$ case, $d_{aggl}\approx 22$, is slightly bigger than the one previously reported in Ref. [15], for which $d_{aggl}\approx 21$ nm. This is due to the larger $M_{S}$ value used here, which enhances the diffusion time (through the diffusion distance, Equation (5)). Nevertheless, the great similarity despite the different $M_{S}$ values emphasizes the key role of the anisotropy in the agglomeration likelihood. The fact that so far we are not considering a nonmagnetic coating has a minor effect, as discussed next.

**Figure 2:**
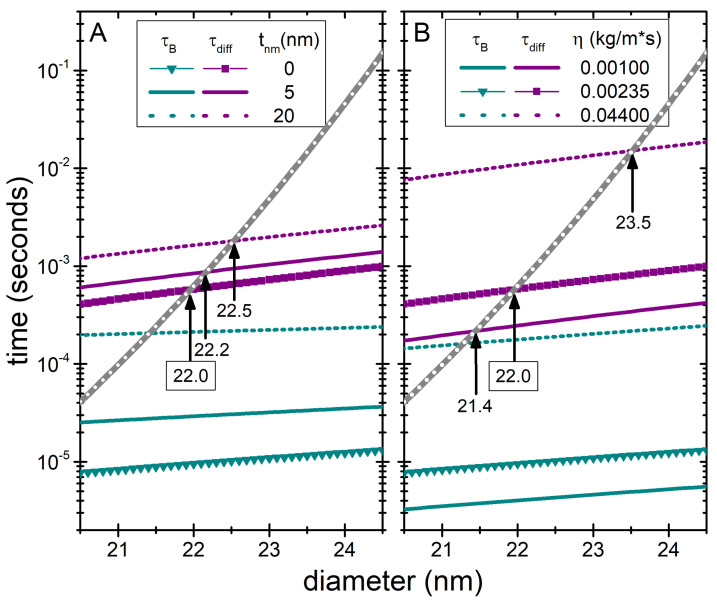
Diffusion ($\tau_{diff}$; purple line), rotation ($\tau_{B}$; green line), and Néel relaxation time ($\tau_{N}$; grey line), as a function of the particle diameter, as in Figure 1, but considering different thickness of the nonmagnetic coating (**A**), or viscosity of the medium (**B**). For simplicity, the results are focused on the K=11 kJ/m${}^{3}$ and the original curves from Figure 1 are reproduced for guidance. The variations of $t_{nm}$ and η are shown with solid and dotted lines, for the values displayed within each panel. The arrows and attached numbers indicate $t_{aggl}$, with the reference one (22.0 nm) highlighted.

While we considered $t_{nm}=0$ in order to determine the boundary where clustering might appear, biomedical applications will always require a biocompatible nonmagnetic coating and therefore it is important to consider its role. That being said, the analysis shows that including a non-magnetic coating does not significantly modify the obtained threshold values: if considering $t_{nm}=5$ nm, $d_{aggl}$ increases just by ∼0.2 nm; and by ∼0.5 nm if $t_{nm}=20$ nm. This is illustrated in Figure 2A.

A slightly larger influence is that of the viscosity of the embedding media, as illustrated in Figure 2B. Considering for example that of water, η=0.001 kg/m·s, it is observed a 0.6 nm decrease from the average size. This value of viscosity is very significant because of being very similar to that of the cell’s cytoplasm, although it must be kept in mind that large variations can be observed within the same cell type and among different types of cells [31]. A much higher viscosity would have a more significant effect, as illustrated for example with the macroscopic value of HeLa cells, η=0.044 kg/m·s; nevertheless this values would be unrealistically high for the current particles, as such large η would correspond to much bigger sizes (over ∼86 nm for HeLa cells) because of the size-dependent viscosity at the microscale [27].

It is important to note that for the anisotropy values considered here, in all cases the size threshold $d_{aggl}$ is always defined by the competition between diffusion and Néel times, as $\tau_{B}<\tau_{diff}$ for all cases shown in Figure 2.

Next we will compare the predictions from the relaxation times with those obtained from *zero field cooling/field cooling* (ZFC/FC) measurements, the common way to estimate SPM behaviour (and thus likely non-agglomeration). Thus, if associating the onset of SPM behaviour to the blocking temperature, estimated as $T_{B}=KV/25k_{B}$[32], the corresponding threshold size, $d_{T_{B}}$, is readily obtained. The comparison between the agglomeration thresholds predicted by both approaches at room temperature (i.e., setting $T_{B}=300$ K) is summarized in Table 1.

Table 1 shows that, on average, the ZFC/FC approach predicts agglomeration to occur for sizes ∼4.2 nm bigger than the ones predicted by the relaxation times approach. In fact, the obtained $d_{T_{B}}$ values correspond to a lower boundary, as they were estimated considering the limit case of no applied field, which is not possible in real ZFC/FC experiments. In general, applying the field during the measurements will result in lower $T_{B}$[33,34,35], which would correspond to larger $d_{T_{B}}$ (at least for the monodisperse case considered here; polydispersity might result in more complex scenarios [36,37,38]).

### 2.2. Associated Heating Performance

**Figure 3:**
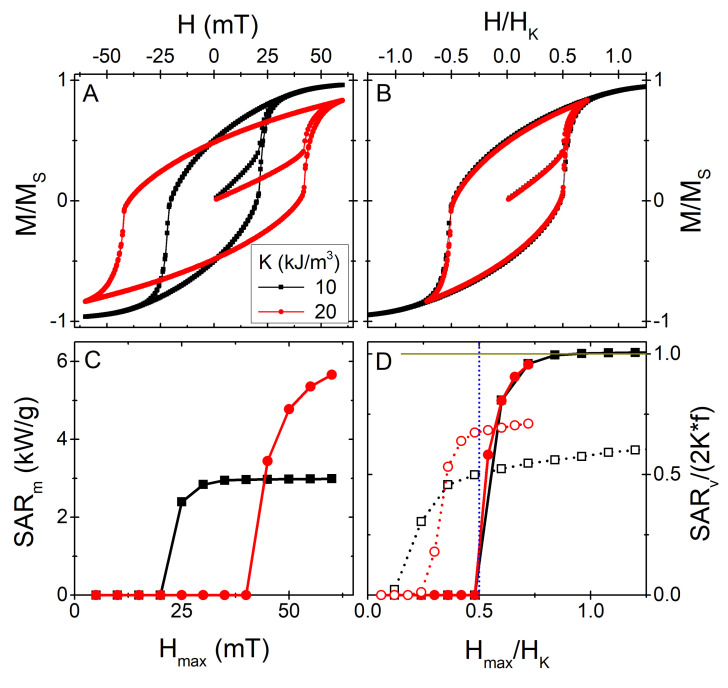
(**A**): Illustrative *Mvs.H* hysteresis loops of two systems of particles of the same size (d=20 nm) and $M_{S}=480$ kA/m, but different *K* (10 and 20 kJ/m${}^{3}$, respectively), at T=0 K and for $H_{max}=25$ mT. (**B**): Same data as in (**A**), replotted in terms of $H/H_{K}$. (**C**): SAR vs. $H_{max}$ for the two different particles, for f=765 kHz, at T=0 K. (**D**): Same data as in panel (**C**), replotted in terms of SAR/2K*f and $H/H_{K}$; the curves with open symbols correspond to the T=300 K case. The vertical blue dotted line stands for the ∼$0.5H_{K}$ threshold of the random distribution [39], and the horizontal solid dark-yellow line indicates the normalized maximum SAR/(2K*f) = 1 limit case.

Similar to its importance on the agglomeration likelihood, the anisotropy plays a principal role in defining the hyperthermia performance. On the one hand, it defines the maximum energy that can be dissipated [4,39]: it is easy to see that for aligned easy axes the maximum hysteresis losses *per loop* are 8K[40] (2K for the random easy axes distribution [24]). On the other hand, it settles the response to the applied field (of amplitude $H_{max}$) through the anisotropy field, defined as $H_{K}=2K/\mu_{0}M_{S}$[25,39]. This double key-role is illustrated in Figure 3, where the heating performance is reported in terms of the usual *Specific Absorption Rate* parameter, SAR, as SAR=A*f, where *A* stands for the area of the loop (hysteresis losses), and *f* is the frequency of the AC field. The simulations were performed in the same way as in Ref. [15]: we considered a random dispersion of monodisperse non-interacting nanoparticles (with the easy axes directions also randomly distributed), and simulated their response under a time varying magnetic field by using the standard Landau-Lifshitz-Gilbert equation of motion within the OOMMF software package [41]; for the random thermal noise (to account for finite temperature) we used the extension module *thetaevolve* [42].

The results displayed in Figure 3 show how, similar to how the apparently different hysteresis loops (A panel) are scaled by the anisotropy field (B panel), the apparently different SAR *vs.* $H_{max}$ trends scale if plotting SAR/(2K*f) vs. $H_{max}/H_{K}$ (the 2f factor is just for normalisation). Note, however, that those results correspond to the Stoner-Wohlfarth-like case at T=0 K [43]. In real systems with finite temperature, *K* also defines—as previously discussed—the stability of the magnetization within the particle. Thus, the ideal T=0 K situation may vary significantly due to the effect of thermal fluctuations, as shown by the open symbols in Figure 3D, which correspond to the T=300 K case for the two particle types considered. It is clearly observed how the strict $H_{max}∼0.5H_{K}$ threshold does not hold, and that the SAR is much smaller than the maximum possible.

The results shown in Figure 3 illustrate well the double role of the anisotropy on the heating performance. What is more, it must be kept in mind that the magnetic anisotropy is the only reason why small particles, such as the ones considered here of typical hyperthermia experiments (well described by the *macrospin* approximation) release heat under the AC field: *if no anisotropy were to exist, there would be no heating* (at least not for the frequencies and fields considered). This applies both to Néel and Brown heating, as with no anisotropy the magnetization would not transfer torque to the particle for its physical reorientation. Of course, larger sizes could display different heating mechanisms (due to non-coherent magnetization behaviour [44] or even eddy currents [45]), but that is not the present case.

**Figure 4:**
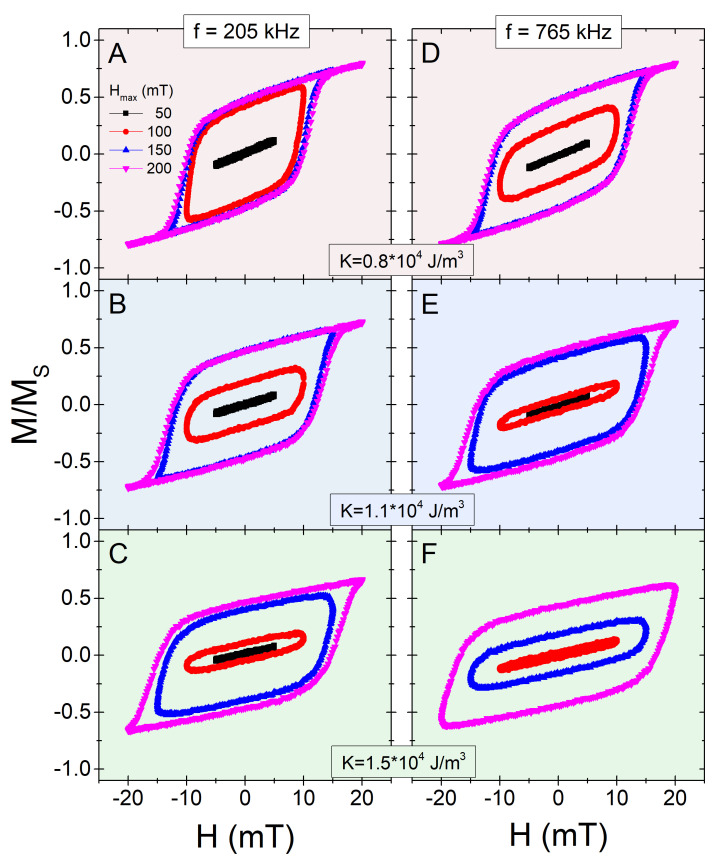
M(H) hysteresis loops, for different $H_{max}$ values, as indicated by the labels in panel (**A**). Left and right columns correspond to f=205 and 765 kHz, respectively. Each pair of colour panels (**A**,**D**), (**B**,**E**), and (**C**,**F**), corresponds to a different *K* value (indicated within the figure) and its corresponding $d_{aggl}$ (Table 1).

We will analyse now the hyperthermia properties of the obtained threshold sizes for the different *K* values. Since the roles of surface coating and media viscosity are not very significant in relation to $d_{aggl}$, we have focused, for simplicity, on the $K-d_{aggl}$ pairs summarized on Table 1, which would set an ideal limit. Thus, we simulated the dynamic hysteresis loops for the three cases considered, to then evaluate the heating capability. Some representative hysteresis loops are shown in Figure 4, for a small (205 kHz) and a large (765 kHz) frequency, as reported in experimental works [11,39,46]. Note that for simplicity we have considered a random easy-axes distribution, but depending on the specific experimental conditions (particle shape and properties of the embedding media, mainly), it might occur as an easy-axes reorientation leading to different hyperthermia performance [46,47,48,49].

**Figure 5:**
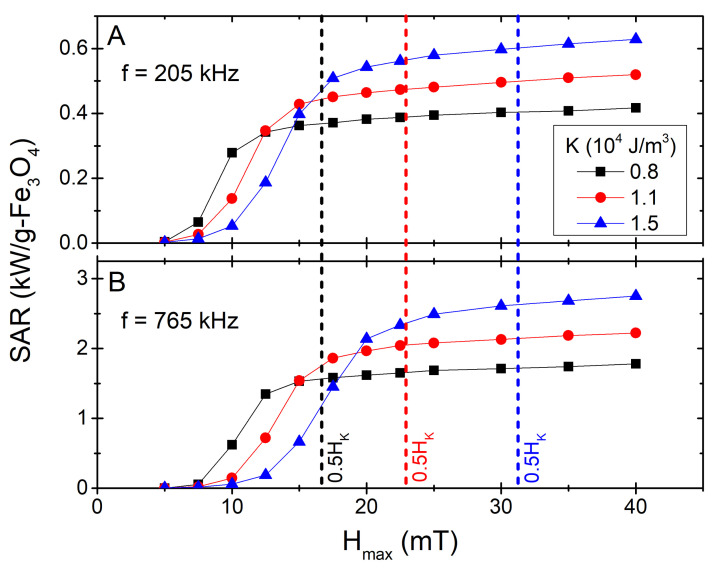
SAR*vs.*$H_{max}$ for the three *K* values (at corresponding $d_{aggl}$), for f=205 and 765 kHz ((**A**) and (**B**) panels, respectively). The vertical lines stand for half of the anisotropy field of each *K* value (of same colour).

The results displayed in Figure 4 show large differences depending on the value of $H_{max}$, illustrative of the minor-major loops competition [24,25]. This is further emphasized by the fact that a higher frequency results in narrower loops for the small fields, but wider for the larger ones. The differences between the different *K* cases are due to the different $H_{max}/H_{K}$ ratios, as discussed in Figure 4. This is systematically analysed through the associated SAR values, shown in Figure 5.

The results plotted in Figure 5 nicely fit within the general scenario discussed previously discussed (Figure 3): larger *K* allows higher SAR, provided enough field amplitude is reached (see corresponding $0.5H_{K}$ values—vertical dashed lines—for reference); for small $H_{max}$ values, however, it may occur that smaller-*K* particles result in higher SAR due to the minor/major loops conditions, as discussed elsewhere [25]. This is an important aspect to consider regarding the variation in *local* heating due to size and/or anisotropy polydispersity [25,50]), as the difference between *blocked* and SPM particles would be the highest and thus also the *locally* released heat [25,51]. The results are also clearly divergent from the *linear response theory* model [19], for which $SAR\propto {H_{max}}^{2}$; this is not surprising as we are far from its applicability conditions (see e.g., Refs. [52,53] for a detailed discussion).

The predicted SAR values are quite large, implying that those particles would make efficient heat mediators. However, it is important to recall here that, so far, we made no considerations to the role of sample concentration. While this may appear reasonable as an initial approach, the fact is that the sample concentration is a key parameter to determine: first, because it defines the amount of deliverable heat; and second, because interparticle interactions (even without agglomeration) may significantly change the heating performance [11,24,29,39,54]. To provide some hint on how the sample concentration, *c* (% volume fraction), relates to the assumptions made, we can consider it through the nearest-neighbors interparticle distance, $l_{NN}$. Following Tewari and Gokhale [55], for a random distribution of monodisperse particles we can approximate $l_{NN}$ as [24]

(6) $$l_{NN}=\left(d+2\cdot t_{nm}\right)\cdot \frac{0.4465}{c^{1/3}}\left[1+1.02625{\left(\frac{c}{0.64}\right)}^{\frac{2}{3}}\right].$$

**Figure 6:**
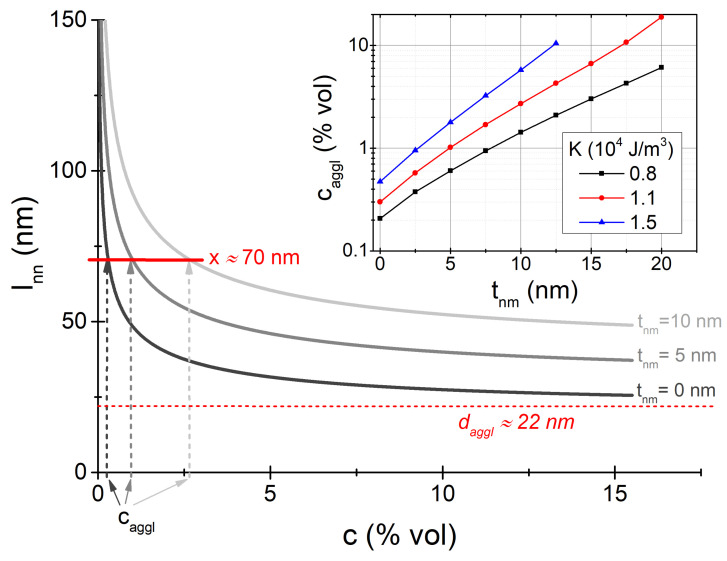
$l_{NN}$*vs. c* curves for different $t_{nm}$ values, for the $K=1.1\times 10^{4}$ J/m${}^{3}$ case. The short (solid) horizontal line indicates the interparticle distance predicted by Equation (5), whereas the long (short-dashed) one indicates the $d_{aggl}$ value, to which $l_{NN}$ tends asymptotically. The vertical arrows indicate the corresponding $c_{aggl}$ values. The inset shows $c_{aggl}$ *vs.* $t_{nm}$ for the different values of *K* considered.

Thus, by equating $l_{NN}$ to the diffusion distance 〈x〉 (Equation (5)) of the different $d_{aggl}$ values, we can obtain the related sample concentration threshold, $c_{aggl}$. This is shown in Figure 6.

The results shown in Figure 6 indicate that for bare particles ($t_{nm}=0$ nm) the applicability of the discussed arguments would be limited to very small concentrations, with $c_{aggl}∼0.2\%$ for the $K=1.1\times 10^{4}$ J/m${}^{3}$ case. However, the presence of a nonmagnetic coating significantly enlarges $c_{aggl}$, as illustrated in the main panel for the cases of $t_{nm}=5$ and 10 nm. This trend is systematically summarized within the inset for the different values of *K*. It is observed that a coating of a few nanometers allows extending the applicability of our arguments within the 1–10% range. It is interesting to notice how with higher *K* this trend occurs with thinner $t_{nm}$, as expected due to the smaller $d_{aggl}$ sizes. At this point it is worth noting that for iron oxides it has been reported the existence of an essentially non-interacting regime at low concentrations [56,57], characteristics that are very attractive for the application viewpoint as it would allow discarding the complex role of interparticle interactions. Nevertheless, it would be clearly interesting to consider the role of interparticle interactions in a more accurate way (e.g., by considering their role on the Néel relaxation time [58]), but unfortunately such an approach is difficult to carry out for a randomly distributed system.

## 3. Conclusions

We have presented an estimation of the threshold sizes for magnetic agglomeration of magnetite-like nanoparticles, depending on their magnetic anisotropy. Our approach was based on the consideration that *K* determines the stability of the particle magnetization and thus the likelihood of magnetic agglomeration, which involves physical translation and rotation of the particles themselves. By comparing the associated timescales, we have obtained that magnetite particles with usual anisotropy values should be relatively stable against agglomeration up to sizes in the range ∼20–25 nm in diameter. Then, we evaluated the associated hyperthermia performance, and found it to be relatively large (hundreds to thousands of W/g) for usual field/frequency conditions. The role of the nonmagnetic surface coating and that of the media viscosity appears secondary in determining the threshold sizes for agglomeration.

The initial estimates were made with no considerations about sample concentration, despite being a critical parameter for the application. In this regard, simple estimates indicate that the assumptions would be strictly valid only for very diluted conditions. However, the presence of a nonmagnetic coating might significantly extend the validity of the approximations to higher concentrations (up to about 10% volume fraction), showing that in this sense the nonmagnetic coating would play a key role.

It is important to recall that we have focused here on purely *magnetic* agglomeration, i.e., an ideal assumption which does not consider the complex situation often found experimentally, where other forces—of electrostatic nature—often play a central role in the agglomeration [59,60,61] and lead to agglomeration at smaller sizes [12]. Including those falls, however, is out of the scope of the present work, as it would result in a scenario that is too complicated. We have neither considered other important system characteristics such as polydispersity in size (both regarding aggregation [15] and heating [50]), and in anisotropy. The latter is expected to play a key role based on its primary importance both for agglomeration and heating, as discussed here. However, to the best of our knowledge its role has only been investigated regarding heating performance [25], but not regarding agglomeration likelihood. Considering the combined influence of those parameters clearly constitutes a challenging task for future works.

Finally, it is necessary to recall the conceptual character of the present work: while we have considered magnetite-like values for *K* and $M_{S}$ as a representative example, for simplicity those were taken as independent of size and temperature. However, it is well known that those may vary significantly within the size range of interest [62], and therefore the accurate determination of the agglomeration likelihood and hyperthermia performance would require including also those dependencies, together with the role of the nonmagnetic coating [63].

References and Note

## Figures and Tables

**Table 1:** Agglomeration size thresholds obtained through the relaxation times approach ($d_{aggl}$) and through the ZFC/FC one ($d_{T_{B}}$), at room temperature for the three anisotropy cases of Figure 1.

*K* (kJ/m${}^{3}$)	$d_{\mathrm{aggl}}$ (nm)	$d_{T_{B}}$ (nm)
8	24.8	29.2
11	22.0	26.2
15	19.5	23.6

## Data Availability

The reported data is available upon reasonable request to the authors.

## References

[1] Wu K., Su D., Liu J., Saha R., Wang J.P. (2019). Magnetic nanoparticles in nanomedicine: A review of recent advances. Nanotechnology.

[2] Colombo M., Carregal-Romero S., Casula M.F., Gutiérrez L., Morales M.P., Bohm I.B., Heverhagen J.T., Prosperi D., Parak W.J. (2012). Biological applications of magnetic nanoparticles. Chem. Soc. Rev..

[3] Ling D., Hyeon T. (2013). Chemical Design of Biocompatible Iron Oxide Nanoparticles for Medical Applications. Small.

[4] Soetaert F., Korangath P., Serantes D., Fiering S., Ivkov R. (2020). Cancer therapy with iron oxide nanoparticles: Agents of thermal and immune therapies. Adv. Drug Deliv. Rev..

[5] Abenojar E.C., Wickramasinghe S., Bas-Concepcion J., Samia A.C.S. (2016). Structural effects on the magnetic hyperthermia properties of iron oxide nanoparticles. Prog. Nat. Sci. Mater. Int..

[6] Fortes Brollo M.E., Domínguez-Bajo A., Tabero A., Domínguez-Arca V., Gisbert V., Prieto G., Johansson C., Garcia R., Villanueva A., Serrano M.C. (2020). Combined Magnetoliposome Formation and Drug Loading in One Step for Efficient Alternating Current-Magnetic Field Remote-Controlled Drug Release. ACS Appl. Mater. Interfaces.

[7] Thorat N.D., Bohara R.A., Noor M.R., Dhamecha D., Soulimane T., Tofail S.A.M. (2017). Effective Cancer Theranostics with Polymer Encapsulated Superparamagnetic Nanoparticles: Combined Effects of Magnetic Hyperthermia and Controlled Drug Release. ACS Biomater. Sci. Eng..

[8] Martel S. (2015). Magnetic nanoparticles in medical nanorobotics. J. Nanopart. Res..

[9] Rojas J.M., Gavilán H., del Dedo V., Lorente-Sorolla E., Sanz-Ortega L., da Silva G.B., Costo R., Perez-Yague S., Talelli M., Marciello M. (2017). Time-course assessment of the aggregation and metabolization of magnetic nanoparticles. Acta Biomater..

[10] Mejías R., Hernández Flores P., Talelli M., Tajada-Herráiz J.L., Brollo M.E., Portilla Y., Morales M.P., Barber D.F. (2019). Cell-Promoted Nanoparticle Aggregation Decreases Nanoparticle-Induced Hyperthermia under an Alternating Magnetic Field Independently of Nanoparticle Coating, Core Size, and Subcellular Localization. ACS Appl. Mater. Interfaces.

[11] Serantes D., Simeonidis K., Angelakeris M., Chubykalo-Fesenko O., Marciello M., Morales M.d.P., Baldomir D., Martinez-Boubeta C. (2014). Multiplying Magnetic Hyperthermia Response by Nanoparticle Assembling. J. Phys. Chem. C.

[12] Gutiérrez L., de la Cueva L., Moros M., Mazarío E., de Bernardo S., de la Fuente J.M., Morales M.P., Salas G. (2019). Aggregation effects on the magnetic properties of iron oxide colloids. Nanotechnology.

[13] Andreu J.S., Camacho J., Faraudo J. (2011). Aggregation of superparamagnetic colloids in magnetic fields: The quest for the equilibrium state. Soft Matter.

[14] Satoh A., Chantrell R.W., Kamiyama S.I., Coverdale G.N. (1996). Three Dimensional Monte Carlo Simulations of Thick Chainlike Clusters Composed of Ferromagnetic Fine Particles. J. Colloid Interf. Sci..

[15] Balakrishnan P.B., Silvestri N., Fernandez-Cabada T., Marinaro F., Fernandes S., Fiorito S., Miscuglio M., Serantes D., Ruta S., Livesey K.L. (2020). Exploiting unique alignment of cobalt ferrite nanoparticles, mild hyperthermia, and controlled intrinsic cobalt toxicity for cancer therapy. Adv. Mater..

[16] Brown W.F. (1963). Thermal Fluctuations of a Single-Domain Particle. Phys. Rev..

[17] Coffey W., Kalmykov Y.P. (2012). The Langevin Equation: With Applications to Stochastic Problems in Physics, Chemistry and Electrical Engineering.

[18] Debye P.J.W. (1929). Polar Molecules.

[19] Rosensweig R. (2002). Heating magnetic fluid with alternating magnetic field. J. Magn. Magn. Mater..

[20] Coffey W.T., Kalmykov Y.P., Titov S.V. (2020). Thermal Fluctuations and Relaxation Processes in Nanomagnets.

[21] Usov N.A. (2010). Low frequency hysteresis loops of superparamagnetic nanoparticles with uniaxial anisotropy. J. Appl. Phys..

[22] Vallejo-Fernandez G., O’Grady K. (2013). Effect of the distribution of anisotropy constants on hysteresis losses for magnetic hyperthermia applications. Appl. Phys. Lett..

[23] Dennis C.L., Krycka K.L., Borchers J.A., Desautels R.D., van Lierop J., Huls N.F., Jackson A.J., Gruettner C., Ivkov R. (2015). Internal Magnetic Structure of Nanoparticles Dominates Time-Dependent Relaxation Processes in a Magnetic Field. Adv. Funct. Mater..

[24] Conde-Leboran I., Baldomir D., Martinez-Boubeta C., Chubykalo-Fesenko O., Morales M.P., Salas G., Cabrera D., Camarero J., Teran F.J., Serantes D. (2015). A Single Picture Explains Diversity of Hyperthermia Response of Magnetic Nanoparticles. J. Phys. Chem. C.

[25] Munoz-Menendez C., Serantes D., Ruso J.M., Baldomir D. (2017). Towards improved magnetic fluid hyperthermia: Major-loops to diminish variations in local heating. Phys. Chem. Chem. Phys..

[26] Gutiérrez L., Costo R., Gruttner C., Westphal F., Gehrke N., Heinke D., Fornara A., Pankhurst Q.A., Johansson C., Veintemillas-Verdaguer S. (2015). Synthesis methods to prepare single- and multi-core iron oxide nanoparticles for biomedical applications. Dalton Trans..

[27] Kalwarczyk T., Ziebacz N., Bielejewska A., Zaboklicka E., Koynov K., Szymański J., Wilk A., Patkowski A., Gapiński J., Butt H.J. (2011). Comparative Analysis of Viscosity of Complex Liquids and Cytoplasm of Mammalian Cells at the Nanoscale. Nano Lett..

[28] Nguyen L., Oanh V., Nam P., Doan D., Truong N., Ca N., Phong P., Hong L., Lam T. (2020). Increase of magnetic hyperthermia efficiency due to optimal size of particles: Theoretical and experimental results. J. Nanopart. Res..

[29] Niculaes D., Lak A., Anyfantis G.C., Marras S., Laslett O., Avugadda S.K., Cassani M., Serantes D., Hovorka O., Chantrell R. (2017). Asymmetric Assembling of Iron Oxide Nanocubes for Improving Magnetic Hyperthermia Performance. ACS Nano.

[30] Santiago-Quinones D., Raj K., Rinaldi C. (2013). A comparison of the magnetorheology of two ferrofluids with different magnetic field-dependent chaining behavior. Rheol. Acta.

[31] Wang K., Sun X.H., Zhang Y., Zhang T., Zheng Y., Wei Y.C., Zhao P., Chen D.Y., Wu H.A., Wang W.H. (2019). Characterization of cytoplasmic viscosity of hundreds of single tumour cells based on micropipette aspiration. R. Soc. Open Sci..

[32] Livesey K.L., Ruta S., Anderson N.R., Baldomir D., Chantrell R.W., Serantes D. (2018). Beyond the blocking model to fit nanoparticle ZFC/FC magnetisation curves. Sci. Rep..

[33] Goya G.F., Morales M.P. (2004). Field Dependence of Blocking Temperature in Magnetite Nanoparticles. J. Metast. Nanocryst. Mater..

[34] Nunes W.C., Socolovsky L.M., Denardin J.C., Cebollada F., Brandl A.L., Knobel M. (2005). Role of magnetic interparticle coupling on the field dependence of the superparamagnetic relaxation time. Phys. Rev. B.

[35] Balaev D., Semenov S., Dubrovskiy A., Yakushkin S., Kirillov V., Martyanov O. (2017). Superparamagnetic blocking of an ensemble of magnetite nanoparticles upon interparticle interactions. J. Magn. Magn. Mater..

[36] Chantrell R.W., Walmsley N., Gore J., Maylin M. (2000). Calculations of the susceptibility of interacting superparamagnetic particles. Phys. Rev. B.

[37] Kachkachi H., Coffey W.T., Crothers D.S.F., Ezzir A., Kennedy E.C., Noguès M., Tronc E. (2000). Field dependence of the temperature at the peak of the zero-field-cooled magnetization. J. Phys. Condens. Matter.

[38] Usov N.A. (2011). Numerical simulation of field-cooled and zero field-cooled processes for assembly of superparamagnetic nanoparticles with uniaxial anisotropy. J. Appl. Phys..

[39] Serantes D., Baldomir D., Martinez-Boubeta C., Simeonidis K., Angelakeris M., Natividad E., Castro M., Mediano A., Chen D.X., Sanchez A. (2010). Influence of dipolar interactions on hyperthermia properties of ferromagnetic particles. J. Appl. Phys..

[40] For a square loop, the coercive field is equal to the anisotropy field, HC=HK. Thus, since HK=2K/MS, the area is A=(2HK)*(2MS)=8K.

[41] Donahue M., Porter D. (2018). OOMMF User’s Guide, Version 1.0, Interagency Report NISTIR 6376.

[42] Lemcke O. (2018). Models Finite Temperature via a Differential Equation of the Langevin Type. http://www.nanoscience.de/group_r/stm-spstm/projects/temperature/download.shtml.

[43] Lacroix L.M., Malaki R.B., Carrey J., Lachaize S., Respaud M., Goya G.F., Chaudret B. (2009). Magnetic hyperthermia in single-domain monodisperse FeCo nanoparticles: Evidences for Stoner–Wohlfarth behavior and large losses. J. Appl. Phys..

[44] Usov N.A., Nesmeyanov M.S., Tarasov V.P. (2018). Magnetic Vortices as Efficient Nano Heaters in Magnetic Nanoparticle Hyperthermia. Sci. Rep..

[45] Morales I., Archilla D., de la Presa P., Hernando A., Marin P. (2020). Colossal heating efficiency via eddy currents in amorphous microwires with nearly zero magnetostriction. Sci. Rep..

[46] Simeonidis K., Morales M.P., Marciello M., Angelakeris M., de La Presa P., Lazaro-Carrillo A., Tabero A., Villanueva A., Chubykalo-Fesenko O., Serantes D. (2016). In-situ particles reorientation during magnetic hyperthermia application: Shape matters twice. Sci. Rep..

[47] Shah S.A., Reeves D.B., Ferguson R.M., Weaver J.B., Krishnan K.M. (2015). Mixed Brownian alignment and Néel rotations in superparamagnetic iron oxide nanoparticle suspensions driven by an ac field. Phys. Rev. B.

[48] Usov N.A., Liubimov B.Y. (2012). Dynamics of magnetic nanoparticle in a viscous liquid: Application to magnetic nanoparticle hyperthermia. J. Appl. Phys..

[49] Mamiya H., Jeyadevan B. (2011). Hyperthermic effects of dissipative structures of magnetic nanoparticles in large alternating magnetic fields. Sci. Rep..

[50] Munoz-Menendez C., Conde-Leboran I., Baldomir D., Chubykalo-Fesenko O., Serantes D. (2015). The role of size polydispersity in magnetic fluid hyperthermia: Average vs. local infra/over-heating effects. Phys. Chem. Chem. Phys..

[51] Aquino V.R.R., Vinícius-Araújo M., Shrivastava N., Sousa M.H., Coaquira J.A.H., Bakuzis A.F. (2019). Role of the Fraction of Blocked Nanoparticles on the Hyperthermia Efficiency of Mn-Based Ferrites at Clinically Relevant Conditions. J. Phys. Chem. C.

[52] Dennis C.L., Ivkov R. (2013). Physics of heat generation using magnetic nanoparticles for hyperthermia. Int. J. Hyperth..

[53] Carrey J., Mehdaoui B., Respaud M. (2011). Simple models for dynamic hysteresis loop calculations of magnetic single-domain nanoparticles: Application to magnetic hyperthermia optimization. J. Appl. Phys..

[54] Branquinho L., Carriao M., Costa A., Zufelato N., Sousa M.H., Miotto R., Ivkov R., Bakuzis A.F. (2013). Effect of magnetic dipolar interactions on nanoparticle heating efficiency: Implications for cancer hyperthermia. Sci. Rep..

[55] Tewari A., Gokhale A. (2004). Nearest-neighbor distances between particles of finite size in three-dimensional uniform random microstructures. Mater. Sci. Eng. C.

[56] Serantes D., Baldomir D., Pereiro M., Hoppe C.E., Rivadulla F., Rivas J. (2010). Nonmonotonic evolution of the blocking temperature in dispersions of superparamagnetic nanoparticles. Phys. Rev. B.

[57] Beola L., Asín L., Roma-Rodrigues C., Fernández-Afonso Y., Fratila R.M., Serantes D., Ruta S., Chantrell R.W., Fernandes A.R., Baptista P.V. (2020). The Intracellular Number of Magnetic Nanoparticles Modulates the Apoptotic Death Pathway after Magnetic Hyperthermia Treatment. ACS Appl. Mater. Interfaces.

[58] Kalmykov Y.P., Titov S.V., Byrne D.J., Coffey W.T., Zarifakis M., Al Bayyari M.H. (2020). Dipole-dipole and exchange interaction effects on the magnetization relaxation of two macrospins: Compared. J. Magn. Magn. Mater..

[59] Faraudo J., Andreu J.S., Camacho J. (2013). Understanding diluted dispersions of superparamagnetic particles under strong magnetic fields: A review of concepts, theory and simulations. Soft Matter.

[60] Bakuzis A.F., Branquinho L.C., Luiz e Castro L., de Amaral e Eloi M.T., Miotto R. (2013). Chain formation and aging process in biocompatible polydisperse ferrofluids: Experimental investigation and Monte Carlo simulations. Adv. Colloid Interface Sci..

[61] Valleau J.P., Ivkov R., Torrie G.M. (1991). Colloid stability: The forces between charged surfaces in an electrolyte. J. Chem. Phys..

[62] Demortiere A., Panissod P., Pichon B.P., Pourroy G., Guillon D., Donnio B., Bégin-Colin S. (2011). Size-dependent properties of magnetic iron oxide nanocrystals. Nanoscale.

[63] Roca A.G., Marco J.F., Morales M.d.P., Serna C.J. (2007). Effect of Nature and Particle Size on Properties of Uniform Magnetite and Maghemite Nanoparticles. J. Phys. Chem. C.

